# *Salacia reticulata* Extract Suppresses Fat Accumulation by Regulating Lipid Metabolism

**DOI:** 10.3390/foods12173149

**Published:** 2023-08-22

**Authors:** Jaeeun Jung, Jeongjin Park, Minhee Lee, Jinhak Kim, Dongchan Oh, Woojin Jun, Ok-Kyung Kim, Jeongmin Lee

**Affiliations:** 1 Department of Medical Nutrition, Kyung Hee University, Yongin 17104, Republic of Korea; jaeeun88@khu.ac.kr (J.J.); miniclsrn@khu.ac.kr (M.L.); 2 Division of Food and Nutrition and Human Ecology Research Institute, Chonnam National University, Gwangju 61186, Republic of Korea; pjj8425@hanmail.net (J.P.); wjjun@jnu.ac.kr (W.J.); 3 R&D Division, Daehan Chemtech Co., Ltd., Seoul 01811, Republic of Korea; jhkim@dhchemtech.com (J.K.); rnd@dhchemtech.com (D.O.)

**Keywords:** *Salacia reticulata*, obesity, lipolysis, adipogenesis, lipogenesis

## Abstract

The excessive storage of triglycerides in adipose tissue is a characteristic feature of obesity, which arises from an imbalance between energy intake and expenditure. In this study, we aimed to explore the potential anti-obesity effects of *Salacia reticulata* extracts (SC) in a high-fat diet (HFD)-induced in obese mice and 3T3-L1 adipocytes, with a specific focus on understanding the underlying lipid mechanisms. Mice were fed with a normal diet (NC; normal control), HFD (60% high-fat diet), Met (HFD containing metformin 250 mg/kg b.w.), SC25 (HFD containing SC 25 mg/kg b.w.), SC50 (HFD containing SC 50 mg/kg b.w.), or SC 100 (HFD containing SC 100 mg/kg b.w.) for 12 weeks. Notably, SC supplementation led to significant reductions in body weight gain, adipose tissue weight, adipose tissue mass, and adipocyte size in HFD-fed mice. Furthermore, SC supplementation exerted inhibitory effects on the adipogenesis and lipogenesis pathways while promoting lipolysis and thermogenesis pathways in the adipose tissues of HFD-fed mice. In vitro experiments using 3T3-L1 cells demonstrated that SC treatment during the differentiation phase suppressed adipogenesis and lipogenesis, whereas SC treatment after differentiation, activated lipolysis and thermogenesis. Collectively, these findings indicate that SC exhibits a direct influence on the lipid metabolism of adipocytes, making it an effective candidate for weight loss interventions.

## 1. Introduction

Obesity and metabolic syndrome are multifactorial conditions influenced by a range of factors, including diet, physical activity, environment, and genetics. It is well known that consistent consumption of high-energy foods, such as snacks, sugar-sweetened drinks, fast foods, and chocolates, are linked to higher rates of metabolic syndrome and obesity [1,2]. Obesity is also characterized by a chronic low-grade inflammatory state, which is linked to the dysregulation of adipokines, cytokines, and other signaling molecules produced by adipose tissue. This inflammation contributes to the development of obesity-related comorbidities, such as type 2 diabetes, cardiovascular disease, and certain types of cancer [3,4,5].

Obesity is a complex metabolic disorder that involves the dysregulation of lipid metabolism, including adipogenesis, lipogenesis, lipolysis, and thermogenesis, which ultimately leads to the accumulation of excessive body fat [6]. In individuals with obesity, adipogenesis and lipogenesis are promoted, resulting in the differentiation of preadipocytes into mature adipocytes and the biosynthesis of fatty acids and triglycerides, ultimately leading to fat accumulation. On the other hand, lipolysis is the breakdown of stored fats into fatty acids for energy, which occurs when energy intake is insufficient to meet the body’s needs, while thermogenesis is the process by which the body generates heat and energy expenditure, playing a crucial role in regulating body weight. The dysregulation of these processes results in the development of obesity. The accumulation of excessive body fat is a consequence of adipocyte hypertrophy and hyperplasia, increased lipogenesis, and decreased lipolysis and thermogenesis [6,7,8,9].

*Salacia reticulata* is a plant species native to India and Sri Lanka that has been used in traditional medicine for centuries [10]. This plant was shown to have anti-inflammatory, antioxidant, antidiabetic, and hypolipidemic properties, positioning it as a promising candidate for addressing metabolic disorders [11,12,13].

Numerous research endeavors have explored the effects of *Salacia reticulata* on obesity, including its ability to reduce body weight, improve lipid metabolism, and regulate adipogenesis [14,15,16]. While *Salacia reticulata* has attracted attention as a natural alternative to traditional pharmacological therapies for treating obesity, the precise mechanism of action has not been elucidated. The aim of the study was to explore the effects of *Salacia reticulata* extract on in high-fat diet (HFD)-induced obese mice and 3T3-L1 adipocytes, with a focus on its underlying biochemical mechanisms.

## 2. Materials and Methods

### 2.1. Preparation of Salacia Reticulata

The extract of *Salacia reticulata* was provided by DAEHAN CHEMTECH Co., Ltd. (Seoul, Republic of Korea). The roots of *Salacia reticulata* were cleaned and pulverized. The dried *Salacia reticulata* roots were extracted with 70% ethanol for a duration of 4 h. The extracted solution was then filtered, concentrated, dried, sieved, and blended (SC).

### 2.2. Obese Mice Model

C57BL/6J male mice (4 weeks old) were procured from Saeron Bio (Uiwang, Republic of Korea) and accommodated in cages with controlled settings (22 ± 2 °C, 55% humidity, and a 12:12 h light-dark cycle). The mice were granted one week to acclimate to these conditions. Mice were fed with a normal diet (NC; normal control, AIN93G diet, Research diet D10012G), HFD (60% high-fat diet, Research diet D12492), Met [HFD containing metformin 250 mg/kg body weight (b.w.)], SC25 (HFD containing SC 25 mg/kg b.w.), SC50 (HFD containing SC 50 mg/kg b.w.), or SC 100 (HFD containing SC 100 mg/kg b.w.) for 12 weeks. Mice were fed with ad libitum access to diet and fresh water and with water consumption averaging 3.2 ± 2.7 mL/day, leading to no significant differences among the groups. The experimental protocol was approved by the Institutional Animal Care and Use Committee of Kyung Hee University (KHGASP-21-117).

### 2.3. Micro-CT

After the mice were anesthetized, whole and abdominal tomographic scans were performed under optimized conditions (Voxel size; 150 μM, energy; 45 kvp, intensity; 110 μA, FOV/Diameter; 79.8 mm, integration; 160 ms) using micro-CT equipment (VIVA CT 80, Scano Medical AG, Wangen-Brüttisellen, Switzerland). The subsequent analysis was performed using the micro-CT Evaluation Program V6.6. During the analysis, the Hounsfield Unit Threshold was configured at values of −200, −30, and 190, and fat measurement was conducted within the HU range of −200 to −30.

### 2.4. Hematoxylin and Eosin (H&E) Staining

Epididymal adipose tissues from mice were fixed with 10% neutral buffered formaldehyde solution to preserve their structure. The fixed sample was then dehydrated with graded ethanol (decreasing concentrations of 100–70%), and embedded in paraffin wax. The paraffin blocks were sliced into 5 μm sections, stained with H&E, washed with dH_2_O, and observed using an optical microscope to visualize adipocytes.

### 2.5. Biochemical Analysis

The levels of triglyceride, total cholesterol, low-density lipoprotein (LDL)/high-density lipoprotein (HDL)-cholesterol, aspartate aminotransferase (AST), and alanine aminotransferase (ALT) were determined in the serum or feces using the instructions provided by the manufacturers of the respective assay kits: Triglyceride Quantification Kit and Cholesterol Assay Kit from Biomax (Seoul, Republic of Korea) and Aspartate Aminotransferase Activity Colorimetric Assay Kit and Alanine Aminotransferase Activity Colorimetric Assay Kit from Biovision.

### 2.6. T3-L1 Cell Culture and Treatment

**Figure 1:**
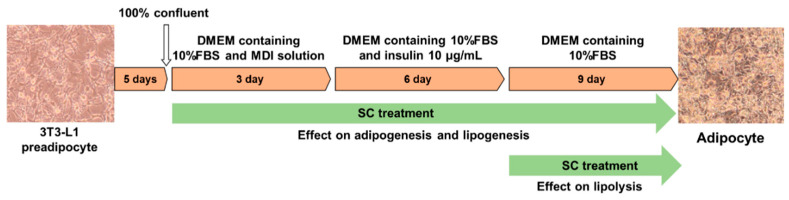
Experimental schematic of differentiation into mature adipocytes and treatment of SC.

The American Type Culture Collection (Rockville, MD, USA) provided the 3T3-L1 preadipocyte cell line, which was cultured under appropriate conditions (95% air, 5% CO_2_, and 37 °C). High-glucose Dulbecco’s Modified Eagle Medium (DMEM; Hyclone Laboratories, Logan, UT, USA) supplemented with 10% newborn calf serum (NCS; Hyclone Laboratories), 1% penicillin/streptomycin (P/S; Hyclone Laboratories), 1% L-glutamine (Hyclone Laboratories), and a 1% sodium pyruvate mixture (Hyclone Laboratories) was used to cultivate the cells. Differentiation into mature adipocytes was performed following the methods described in Figure 1 using an adipogenic cocktail (0.5 mM 3-isobutyl-1-methylxanthine, 10 μg/mL insulin, and 1 μM dexamethasone; Sigma-Aldrich, St. Louis, MO, USA).

### 2.7. Oil Red O Staining

After washing the cells with DPBS, they were fixed in a 10% formalin solution and the plate was dried with 60% isopropanol. Lipid droplets were stained with the Oil Red O working solution for 2 h, followed by washing with distilled water 4 times and photographing. To quantify lipid accumulation, 100% isopropanol was added to elute the Oil Red O dye from the plate.

### 2.8. Western Blot

Proteins were extracted from the epididymal adipose tissues and brown adipose tissues of mice and cells were lysed using a lysis reagent (Sigma, St. Louis, MO, USA), while an equal amount of protein was separated using a 10% MiniPROTEAN^®^ TGX™ Precast Protein Gel (Bio-Rad Laboratories, Hercules, CA, United States). The separated proteins were transferred electrophoretically onto membranes using the Trans-Blot^®^ TurboTM Transfer system (Bio-Rad). The membranes were then blocked and incubated with primary antibodies against phosphorylated mitogen-activated protein kinase (MAPK), sterol regulatory element-binding protein (SREBP) 1, CCAAT/enhancer binding protein (C/EBP) α, peroxisome proliferator-activated receptor (PPAR)-γ, glucose-6-phosphate dehydrogenase (G6PDH), phosphorylated ATP-citrate lyase (ACL), ACL, phosphorylated acetyl-CoA carboxylase (ACC), ACC, fatty acid synthase (FAS), lipoprotein lipase (LPL), adiponectin, leptin, protein kinase A (PKA), phosphodiesterase 3B (PDE3B), p-HSL, HSL, perilipin, adipose triglyceride lipase (ATGL), phosphorylated AMP-activated protein kinase (AMPK), AMPK, FABP4, uncoupling protein-1 (UCP-1), carnitine palmitoyltransferase 1 (CPT1), and β-actin (Cell Signaling, Beverly, MA, USA), before being subsequently incubated with a secondary antibody (anti-rabbit IgG HRP-linked antibody, 1:5000, Cell Signaling). The resulting protein bands were detected, quantified using the CS Analyzer 3.0 (ATTO), and normalized using β-actin as a loading control.

### 2.9. Measurement of cAMP Levels

The cAMP levels in the white adipose tissue (WAT) of mice and in cells were measured using an ELISA kit from Cell biolabs Inc., (San Diego, CA, USA). The manufacturer’s manual was followed during the experiment.

### 2.10. Statistical Analysis

The mean ± SD was used to present all data. Statistical analysis was performed using one-way ANOVA, and multiple comparisons were conducted using Duncan’s multiple range test. SPSS PASW Statistic v.23.0 (SPSS Inc., Chicago, IL, USA) was used for statistical analysis, and *p* < 0.05 was considered statistically significant.

## 3. Results

### 3.1. SC Supplementation Prevented HFD-Induced Obesity

The HFD group showed a significant increase in body weight gain, food efficiency ratio, and liver and adipose tissue weights compared to the normal control group. Dietary supplementation with SC led to a significant decrease in body weight gain, food efficiency ratio, and liver and adipose tissues weights of mice fed a HFD compared to the HFD groups, indicating that SC supplementation effectively prevented HFD-induced obesity (Table 1) (*p* < 0.05).

**Figure 2:**
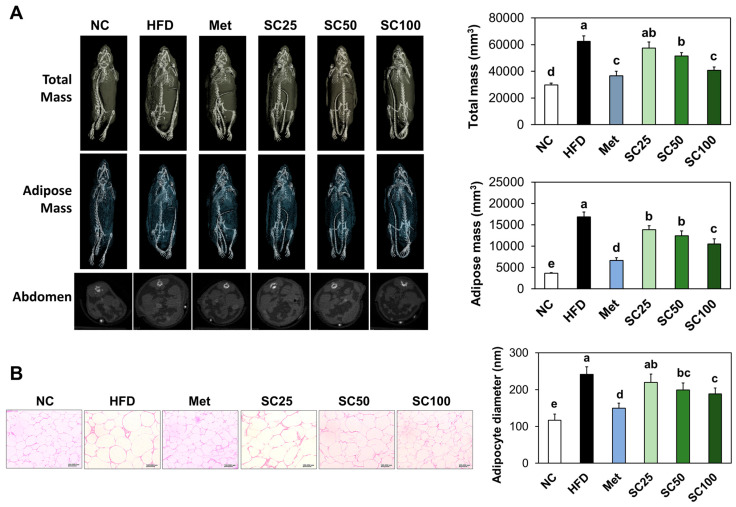
Effects of *Salacia reticulata* extracts on adipose tissue mass (**A**) and lipid drops size (**B**) in the adipose tissue in HFD-induced obese mice. NC, normal control; HFD, 60% high-fat diet; HFD+Met, Metformin 250 mg/kg BW in 60% high-fat diet; SC, *Salacia reticulata* extracts 25 mg/kg BW in 60% high-fat diet; SC50, *Salacia reticulata* extracts 50 mg/kg BW in 60% high-fat diet; SC100, *Salacia reticulata* extracts 100 mg/kg BW in 60% high-fat diet. Values are presented as mean ± standard deviation (*n* = 8), and different superscript letters indicate significance at *p* < 0.05.

We observed a significant increase in adipose tissue mass in HFD-fed mice compared to those in the normal diet-fed mice (Figure 2A). Additionally, mice of HFD group had a significantly larger adipocyte size compared to that in the normal control group (Figure 2B). However, supplementation with SC significantly reduced adipose mass and lipid drops production relative to the HFD group (Figure 2) (*p* < 0.05).

Supplementation with HFD resulted in elevated levels of serum triglycerides, total cholesterol, LDL, HDL, ALT, and AST, and fecal total cholesterol and triglycerides increased significantly compared with those of the normal diet. The Met and SC groups exhibited a significant decrease in the levels of serum triglycerides, total cholesterol, LDL cholesterol, HDL cholesterol, ALT, and AST compared with the HFD group. Moreover, SC supplementation induced significant increases in fecal levels of total cholesterol and triglycerides (Table 2) (*p* < 0.05).

### 3.2. SC Supplementation Suppressed Adipogenesis and Lipogenesis in HFD-Fed Mice

**Figure 3:**
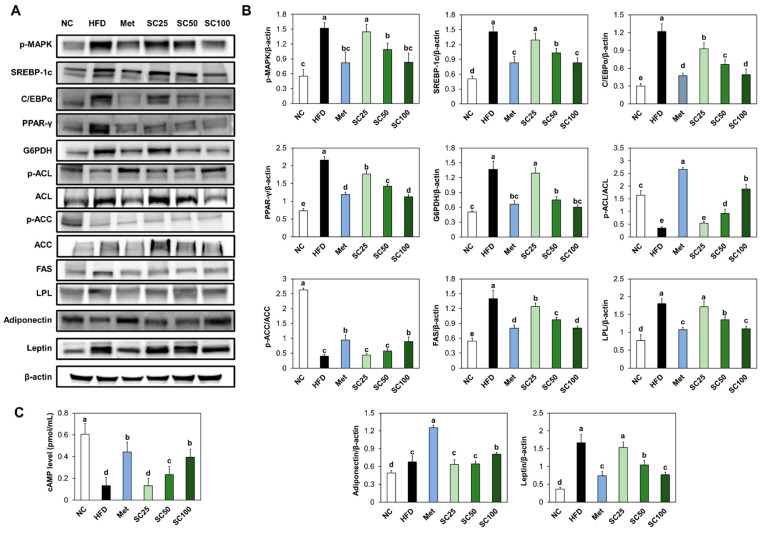
Effects of *Salacia reticulata* extracts on the expression of protein-related adipogenesis and lipogenesis ((**A**) band image; (**B**) quantification) and levels of cAMP (**C**) in white adipose tissue from HFD-induced obese mice. NC, normal control; HFD, 60% high-fat diet; Met, Metformin 250 mg/kg BW in 60% high-fat diet; SC, *Salacia reticulata* extracts 25 mg/kg BW in 60% high-fat diet; SC50, *Salacia reticulata* extracts 50 mg/kg BW in 60% high-fat diet; SC100, *Salacia reticulata* extracts 100 mg/kg BW in 60% high-fat diet. Values are presented as mean ± standard deviation (*n* = 8), and different superscript letters indicate significance at *p* < 0.05.

Compared to the normal control group, the expression levels of phosphorylated MAPK, adipogenic transcription factors (SREBP-1c, C/EBP α, and PPAR-γ), and G6PDH were significantly increased in the HFD group (Figure 3A,B). Furthermore, we observed a significant increase in the levels of dephosphorylated ACL and ACC, FAS, and LPL, accompanied by a decrease in cAMP levels in the white adipose tissue of HFD-fed mice, in comparison to those of the normal mice (Figure 3A–C). However, SC supplementation effectively suppressed these changes in adipogenesis and lipogenesis factors induced by HFD (Figure 3A–C). Additionally, we found that SC supplementation in HFD-fed mice stimulated the protein expression of adiponectin and suppressed the protein expression of leptin, compared to the HFD group (Figure 3A,B) (*p* < 0.05).

### 3.3. SC Supplementation Stimulated Lipolysis and Thermogenesis in HFD-Fed Mice

**Figure 4:**
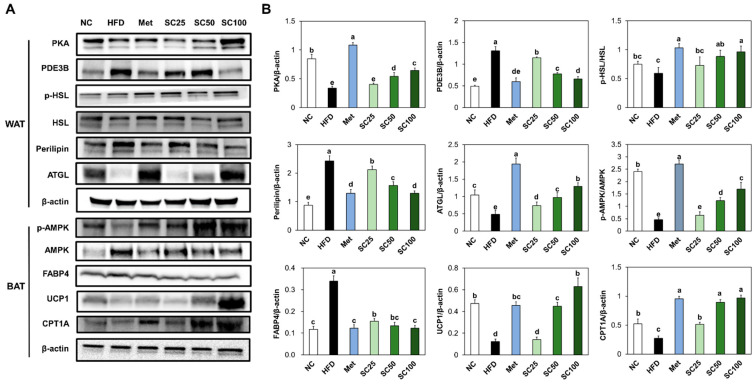
Effects of *Salacia reticulata* extracts on the expression of protein-related lipolysis and thermogenesis ((**A**) band image; (**B**) quantification) and levels of cAMP in white adipose tissue or brown adipose tissue from HFD-induced obese mice. NC, normal control; HFD, 60% high-fat diet; Met, Metformin 250 mg/kg BW in 60% high-fat diet; SC, *Salacia reticulata* extracts 25 mg/kg BW in 60% high-fat diet; SC50, *Salacia reticulata* extracts 50 mg/kg BW in 60% high-fat diet; SC100, *Salacia reticulata* extracts 100 mg/kg BW in 60% high-fat diet. Values are presented as mean ± standard deviation (*n* = 8), and different superscript letters indicate significance at *p* < 0.05.

The protein expressions of PKA and ATGL were notably reduced, whereas PDE3B and perilipin showed a significant increase in the white adipose tissue from the HFD group, as compared to the normal control. In brown adipose tissue (BAT), thermogenesis-related proteins, including phosphorylated AMPK, UCP-1, and CPT1, were significantly decreased in the HFD group compared to the normal control. However, supplementation with SC activated the lipolysis and thermogenesis pathway compared to the HFD group (Figure 4) (*p* < 0.05).

### 3.4. SC Treatment Suppressed Adipogenesis and Lipogenesis in Adipocytes

**Figure 5:**
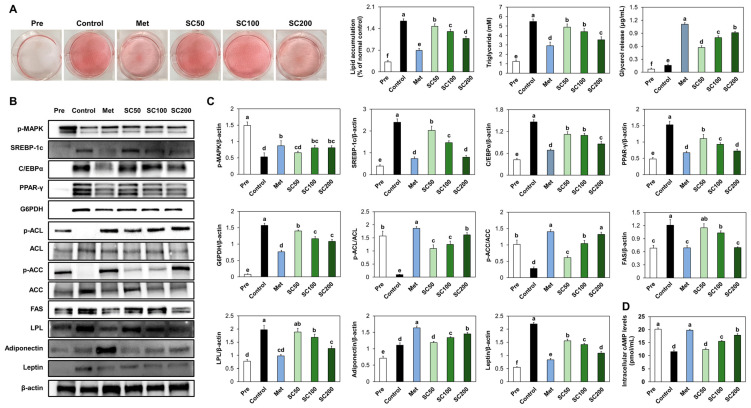
Impact of SC treatment on the triglyceride accumulation (**A**), adipogenesis and lipogenesis ((**B**) band image; (**C**) quantification), and levels of cAMP (**D**) in 3T3-L1 cells. Pre, undifferentiated preadipocyte 3T3-L1 cells; Control, differentiated 3T3-L1 cells; Met, metformin treatment at 1 mM; SC50, SC treatment at 50 μg/mL; SC100, SC treatment at 100 μg/mL; SC200, SC treatment at 200 μg/mL. Values are presented as mean ± standard deviation (*n* = 3), and different superscript letters indicate significance at *p* < 0.05.

In our study, we observed that the treatment of SC during the differentiation of 3T3-L1 cells resulted in a reduction of intracellular triglyceride levels, as compared to the control cells (Figure 5A). Additionally, the expression levels of proteins involved in adipogenesis and lipogenesis pathways were significantly reduced and intracellular cAMP levels were significantly increased in SC-treated 3T3-L1 cells compared to the control cells (Figure 5B–D).

### 3.5. SC Treatment Stimulated Lipolysis and Thermogenesis in Adipocytes

**Figure 6:**
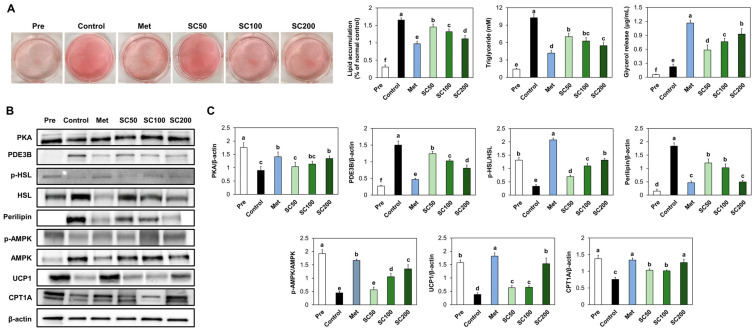
Impact of SC treatment on the triglyceride accumulation (**A**) and lipolysis and thermogenesis ((**B**) band image; (**C**) quantification) in 3T3-L1 cells after differentiation. Pre, undifferentiated preadipocyte 3T3-L1 cells; Control, differentiated 3T3-L1 cells; Met, metformin treatment at 1 mM; SC50, SC treatment at 50 μg/mL; SC100, SC treatment at 100 μg/mL; SC200, SC treatment at 200 μg/mL. Values are presented as mean ± standard deviation (*n* = 3), and different superscript letters indicate significance at *p* < 0.05.

To confirm the stimulatory effects of SC on lipolysis and thermogenesis, we examined the intracellular triglyceride, as well as the lipid metabolism, in differentiated 3T3-L1 cells. Our findings revealed that SC treatment in adipocytes suppressed intracellular triglyceride (Figure 6A) and stimulated the lipolysis and thermogenesis-related factors compared to the control cells (Figure 6B,C).

## 4. Discussion

Previous research papers on *Salacia reticulata* suggest that its extract exhibits potential anti-obesity effects by reducing body weight and inhibiting adipocytes differentiation [14,15,16]. The study by Kishino et al. [14] conducted on obese mice supplemented with mixture of the *Salacia reticulata* aqueous extract and cyclodextrin, found a significant reduction in body weight compared to the control group. The extract was shown to effectively suppress weight gain and decrease fat accumulation. In addition, Shimada et al. [16] demonstrated that concurrent treatment of *Salacia reticulata* extract and differentiation inducers significantly inhibited the differentiation process, preventing the maturation of adipocytes. While these studies provide promising results regarding the anti-obesity effects of *Salacia reticulata*, it is important to note that further research is still needed to better understand the mechanisms of action, optimal dosage, and potential side effects *of Salacia reticulata* extract. The current study determined the effect of *Salacia reticulata* on mechanisms related to lipid metabolism using obese mice and 3T3-L1 cells.

In the adipocyte differentiation, MAPK pathways regulate adipogenesis at each steps of the process by stimulation the expression of adipogenesis-related factors [17]. Our findings demonstrated that HFD induced the upregulation of phosphorylated MAPK as well as adipogenesis-related transcription factors in the WAT of mice and 3T3-L1 cells. Both SC supplementation in HFD-fed mice and SC treatment in 3T3-L1 adipocytes resulted in reduced protein expression of phosphorylated MAPK and adipogenesis-related transcription factors, suggesting the inhibitory effect of SC on adipocyte differentiation.

De novo lipogenesis primarily occurs in the liver and adipose tissue, particularly in response to an excess of dietary energy. The process of de novo lipogenesis begins with the conversion of glucose or other carbohydrates into acetyl-CoA, a key molecule in cellular metabolism. This conversion is facilitated by enzymes such as ACL and ACC. Once acetyl-CoA is generated, the rate-limiting step in de novo lipogenesis process is catalyzed by the enzyme FAS. The newly synthesized triglycerides can be stored within adipocytes, specialized cells that store fat, or transported to other tissues for utilization as an energy source when needed. Excessive de novo lipogenesis can contribute to increased fat accumulation and obesity [18,19]. Our results indicated that supplementing SC resulted in decreased activations of lipogenesis-related factors. These results strongly suggest that SC displays anti-obesity activity by curbing fat accumulation through the inhibition of both adipogenesis and lipogenesis processes.

Leptin and adiponectin are adipokines, which are hormones secreted by adipose tissue and play important roles in modulating lipid metabolism and energy homeostasis. Leptin functions as a satiety hormone and plays a key role in regulating body weight and energy balance. When adipose tissue mass increases, leptin levels rise, and this increase in leptin acts on the hypothalamus to suppress appetite and increase energy expenditure. When abnormal fat accumulation occurs in adipose tissue, excessive leptin production may lead to leptin resistance and a loss of appetite control. Adiponectin plays a crucial role in regulating insulin sensitivity and lipid metabolism. It enhances insulin sensitivity in peripheral tissues, such as skeletal muscle and liver, leading to increased fatty acid oxidation (fat burning) and decreased triglyceride synthesis. Adiponectin also acts on the liver to suppress the expression of key enzymes involved in de novo lipogenesis, such as ACC and FAS. Disruptions in leptin and adiponectin signaling can lead to dysregulation of lipid metabolism and contribute to the development of metabolic disorders [20,21]. Remarkably, our study revealed that supplementation with SC effectively reduced the levels of leptin and increased the levels of adiponetin. These findings indicate that SC supplementation has the potential to decrease leptin secretion and prevent the development of leptin resistance by mitigating adipocyte hypertrophy.

Adipocytes catalyze TG to produce fatty acids, which are then metabolized through β-oxidation to generate ATP and facilitate the tricarboxylic acid TCA cycle [22,23]. In our study, we observed that SC supplementation resulted in the activation of lipolysis and thermogenesis in both HFD-fed mice and 3T3-L1 adipocytes. BAT primarily serves the important role of engaging in thermogenesis, a process that converts energy into heat. This thermogenic activity is regulated by AMPK, a key regulator of energy metabolism [24,25]. The study by Shimada et al. [15] also demonstrated that treatment with *Salacia reticulata* extract in mature 3T3-L1 cells led to the suppression of intracellular triacylglycerol accumulation and enhancement of glycerol release into the medium. Based on these previous studies, as well as our present results, we propose that SC has the potential to enhance energy expenditure through the stimulation of lipolysis and thermogenesis processes.

In this study, we confirmed that SC exhibits dual effects by inhibiting adipogenesis while promoting lipolysis. Further mechanistic molecular investigations are required to validate that these multifaceted changes were induced, rather than solely attributed to alterations in a specific intracellular factor. Additionally, it is important to note that while these results are promising, translating them into viable weight loss strategies for human application necessitates extensive clinical trials. Notably, the specific active compound responsible for these effects in SC remains unidentified in our study, underscoring the need for further research in this direction.

## 5. Conclusions

Our findings clearly indicate that supplementation with SC resulted in substantial decreases in body weight gain, adipose mass, and adipocyte size among obese mice. Additionally, SC inhibited the adipogenesis and lipogenesis pathways while enhancing the lipolysis and thermogenesis pathways in both obese mice and adipocytes, indicating the potential of SC supplementation to directly suppress fat accumulation by regulating lipid metabolism in adipocytes. These findings provide scientific evidence supporting the weight loss properties of SC and elucidate the fundamental mechanisms contributing to its anti-obesity effects.

## Figures and Tables

**Table 1:** Effects of *Salacia reticulata* extracts on body weight, weight gain, and organ and adipose tissue weight in mice with HFD-induced obese mice.

	NC	HFD	Met	SC25	SC50	SC100
Initial body weight (g)	21.68 ± 1.82	19.85 ± 2.92	20.42 ± 1.56	19.17 ± 3.03	19.07 ± 32.4	19.45 ± 1.47
Final body weight (g)	31.30 ± 3.68 ^e^	50.47 ± 2.61 ^a^	40.89 ± 3.10 ^d^	48.27 ± 2.45 ^ab^	45.99 ± 1.92 ^bc^	43.67 ± 2.81 ^cd^
Weight gain (g)	9.62 ± 2.88 ^e^	30.62 ± 3.61 ^a^	20.47 ± 1.72 ^d^	28.84 ± 2.56 ^ab^	26.92 ± 3.17 ^bc^	24.22 ± 1.59 ^c^
Food intake (kcal/day)	9.02 ± 0.17 ^c^	14.13 ± 1.05 ^a^	13.23 ± 0.52 ^b^	14.08 ± 1.14 ^a^	13.82 ± 0.64 ^ab^	13.48 ± 0.51 ^ab^
Organ weight (g)						
Liver	1.41 ± 0.14 ^c^	2.17 ± 0.35 ^a^	1.78 ± 0.33 ^b^	1.93 ± 0.29 ^ab^	1.85 ± 0.28 ^ab^	1.77 ± 0.13 ^b^
Kidney	0.33 ± 0.04 ^c^	0.44 ± 0.04 ^a^	0.39 ± 0.03 ^ab^	0.41 ± 0.03 ^ab^	0.40 ± 0.03 ^ab^	0.40 ± 0.03 ^ab^
Spleen	0.09 ± 0.01 ^b^	0.13 ± 0.01 ^a^	0.10 ± 0.01 ^b^	0.11 ± 0.01 ^ab^	0.11 ± 0.02 ^ab^	0.11 ± 0.02 ^b^
Adipose tissue weight (g)						
Total white adipose tissue	1.83 ± 0.49 ^e^	6.43 ± 0.64 ^a^	3.78 ± 0.53 ^d^	5.87 ± 0.54 ^ab^	5.56 ± 0.66 ^b^	4.84 ± 0.68 ^c^
Subcutaneous WAT	0.83 ± 0.25 ^e^	3.05 ± 0.40 ^a^	1.46 ± 0.35 ^d^	2.73 ± 0.37 ^ab^	2.58 ± 0.37 ^bc^	2.19 ± 0.32 ^c^
Visceral WAT	0.26 ± 0.07 ^e^	1.16 ± 0.22 ^a^	0.67 ± 0.10 ^d^	1.03 ± 0.10 ^ab^	0.91 ± 0.17 ^bc^	0.08 ± 0.09 ^cd^
Brown adipose tissue	0.10 ± 0.03 ^d^	0.20 ± 0.04 ^a^	0.11 ± 0.03 ^d^	0.18 ± 0.04 ^ab^	0.16 ± 0.05 ^bc^	0.14 ± 0.03 ^cd^

NC, normal control; HFD, 60% high-fat diet; HFD+Met, Metformin 250 mg/kg BW in 60% high-fat diet; SC, *Salacia reticulata* extracts 25 mg/kg BW in 60% high-fat diet; SC50, *Salacia reticulata* extracts 50 mg/kg BW in 60% high-fat diet; SC100, *Salacia reticulata* extracts 100 mg/kg BW in 60% high-fat diet. Values are presented as mean ± standard deviation (*n* = 8), and different superscript letters indicate significance at *p* < 0.05.

**Table 2:** Effects of *Salacia reticulata* extracts on lipid profiles, ALT and AST levels in mice with high-fat diet induced obesity.

	NC	HFD	Met	SC25	SC50	SC100
Serum						
Total cholesterol (μg/μL)	10.07 ± 1.04 ^e^	20.16 ± 1.62 ^a^	12.05 ± 0.85 ^d^	18.78 ± 1.26 ^ab^	17.41 ± 1.54 ^b^	14.82 ± 1.66 ^c^
Triglyceride (nmol/μL)	8.62 ± 2.42 ^e^	23.38 ± 2.61 ^a^	12.22 ± 1.81 ^d^	22.41 ± 2.52 ^ab^	19.78 ± 2.28 ^b^	16.66 ± 1.89 ^c^
HDL-cholesterol (μg/μL)	109.72 ± 14.60 ^c^	195.09 ± 16.92 ^a^	167.49 ± 14.53 ^b^	174.57 ± 23.72 ^b^	174.20 ± 11.39 ^b^	170.79 ± 15.41 ^b^
LDL-cholesterol (μg/μL)	26.77 ± 6.56 ^e^	74.32 ± 5.12 ^a^	40.95 ± 6.23 ^d^	66.26 ± 6.25 ^b^	57.66 ± 7.75 ^c^	48.54 ± 6.36 ^d^
LDL/HDL ratio	0.24 ± 0.05 ^c^	0.38 ± 0.05 ^a^	0.25 ± 0.04 ^c^	0.38 ± 0.03 ^a^	0.33 ± 0.06 ^ab^	0.28 ± 0.03 ^bc^
ALT (mU/mL)	4.30 ± 2.65 ^d^	19.96 ± 1.99 ^a^	12.42 ± 2.33 ^c^	17.90 ± 2.54 ^ab^	16.31 ± 2.47 ^b^	16.26 ± 2.82 ^b^
AST (mU/mL)	14.66 ± 5.93 ^e^	49.97 ± 5.01 ^a^	27.80 ± 5.19 ^d^	45.89 ± 5.39 ^ab^	42.17 ± 4.09 ^bc^	39.48 ± 2.93 ^c^
Fecal						
Total cholesterol (μg/μL)	7.24 ± 0.73 ^e^	9.55 ± 0.78 ^d^	14.53 ± 1.57 ^a^	9.80 ± 0.78 ^d^	11.20 ± 0.96 ^c^	12.64 ± 1.30 ^b^
Triglyceride (nmol/μL)	5.35 ± 0.59 ^e^	7.88 ± 0.64 ^d^	13.71 ± 0.82 ^a^	7.99 ± 1.13 ^d^	10.82 ± 0.84 ^c^	12.58 ± 0.79 ^b^

NC, normal control; HFD, 60% high-fat diet; Met, Metformin 250 mg/kg BW in 60% high-fat diet; SC, *Salacia reticulata* extracts 25 mg/kg BW in 60% high-fat diet; SC50, *Salacia reticulata* extracts 50 mg/kg BW in 60% high-fat diet; SC100, *Salacia reticulata* extracts 100 mg/kg BW in 60% high-fat diet. Values are presented as mean ± standard deviation (*n* = 8), and different superscript letters indicate significance at *p* < 0.05.

## Data Availability

The data used to support the findings of this study can be made available by the corresponding author upon request.
